# Effect of Reaction Temperature on Structure, Appearance and Bonding Type of Functionalized Graphene Oxide Modified *P*-Phenylene Diamine

**DOI:** 10.3390/ma11040647

**Published:** 2018-04-23

**Authors:** Hong-Juan Sun, Bo Liu, Tong-Jiang Peng, Xiao-Long Zhao

**Affiliations:** 1 Key Laboratory of Ministry of Education for Solid Waste Treatment and Resource Recycle, Southwest University of Science and Technology, Mianyang 621010, China; tjpeng@swust.edu.cn (T.-J.P.); skygraphene@163.com (X.-L.Z.); 2 School of National Defense Science and Technology, Southwest University of Science and Technology, Mianyang 621010, China

**Keywords:** graphene oxide, *p*-phenylene diamine, functionalized graphene oxide, cross-link bond type, bonding type

## Abstract

In this study, graphene oxides with different functionalization degrees were prepared by a facile one-step hydrothermal reflux method at various reaction temperatures using graphene oxide (GO) as starting material and *p*-phenylenediamine (PPD) as the modifier. The effects of reaction temperature on structure, appearance and bonding type of the obtained materials were investigated by X-ray diffraction (XRD), Fourier transform infrared spectroscopy (FT-IR), X-ray photoelectron spectroscopy (XPS), and scanning electron microscopy (SEM). The results showed that when the reaction temperature was 10–70 °C, the GO reacted with PPD through non-covalent ionic bonds (–COO^−^H_3_^+^N–R) and hydrogen bonds (C–OH…H_2_N–X). When the reaction temperature reached 90 °C, the GO was functionalized with PPD through covalent bonds of C–N. The crystal structure of products became more ordered and regular, and the interlayer spacing (*d* value) and surface roughness increased as the temperature increased. Furthermore, the results suggested that PPD was grafted on the surface of GO through covalent bonding by first attacking the carboxyl groups and then the epoxy groups of GO.

## 1. Introduction

Graphene is attracting increasing attention in physics, chemistry and material research due to its unique laminar crystal structure [1], high electrical conductivity [2], high thermal conductivity [3], excellent flexibility, and mechanical properties [4,5,6,7]. The chemical reduction of graphene oxide has been regarded as an effective way to achieve large-scale preparation of grapheme [8,9,10]. However, the addition of only hydrazine hydrates without other materials will likely result in aggregated graphene [11,12]. Therefore, the formation of monolithic and high-performance graphene will require further treatments besides the necessary reduction reaction.

Compared to graphene, graphene oxide (GO) possesses a large number of carboxyl groups (–COOH) near the edges, with many epoxy groups (C–O–C) and hydroxyl groups (C–OH) on the surface. These oxygen-containing groups increase the reaction activity of GO. Therefore, functionalization of GO can be achieved using various approaches, such as through hydrogen, ionic, and covalent bonding [13,14,15]. Hence, the structure of graphene can be regulated in order to modify its light, electrical, and magnetic properties [15,16].

In recent years, various amine based chemicals have been used to modify GO. These include ammonia [17], cetylamine [18], octadecylamine [19], ethidenediamine [20], poly-o-phenylenediamine [21], and amino acids [22]. For instance, Shanmugharaj et al. [23] used alkylamines with different chain lengths as modifiers and mixed them with GO under ultrasonic conditions at normal temperature, followed by suction filtration during the first reaction stage. Next, they subjected the suspension to vacuum drying treatment to yield functionally modified GO. They detected the formation of ionic, hydrogen, and covalent bonds between aliphatic amine and GO. The surface roughness degree of GO increased after modification, and roughness degree rose asalkylamine chain length was extended. Matsuo et al. [24] intercalated alkylamines with different chain lengths into GO, and identified three types of interactions between alkylamines in GO. In addition, the arrangement patterns of alkylamines with different chain lengths were different, yielding various interlamellar spacing. Hung et al. [25] used three amine monomers (ethidenediamine, butanediamine, and p-phenylenediamine) as cross-linking agents to prepare GO skeleton sheets with different interlamellar spacing using pressure-assisted self-assembling technology. During the modification process, amine monomer was combined with GO through chemical bonding to yield GO with significantly changed hydrophobicity. Furthermore, the cross-linking mode changed from non-aromatic cyclamine cross-linking to aromatic cyclamine cross-linking, and the composite film-water contact angle varied from 24.4° to 80.6°. 

According to previous literature [26,27], GO was functionalized with amine by reacting at a temperature over 90 °C. However, the reason for selecting this temperature and the effects of reaction temperature on structure, appearance, and interaction mechanisms of FGO have not been reported so far. In this paper, FGO composites were prepared using a facile one-step hydrothermal reflux method under different reaction temperatures using GO as starting material and PPD as the modifier. The structures, functional groups and compositions of the prepared composites were investigated by XRD, Raman, SEM, FT-IR, and XPS analyses. The effects of reaction temperature on structure and appearance of modified composites were also examined. Finally, the mechanism of interaction between PPD and GO at different reaction temperatures was explored.

## 2. Experimental

### 2.1. Reagents 

The following reagents were used in this study: natural flake graphite (Tangseng Gou, Xinghe County, Inner Mongolia, China, with carbon content ≥90%, screened by 200 mesh), potassium permanganate and concentrated sulfuric acid (Sinopharm, Shanghai, China), H_2_O_2_ solution (5%) and 0.05 mol·L^−1^ HCl solution (Chengdu Jinshan Chemical Reagent, Chengdu, China), *p*-phenylene diamine(PPD), and methyl alcohol (≥99.5%, Chengdu Kelong Chemical Reagent Factory, Chengdu, China). All reagents were of analytical grade. Ultrapure water with a resistivity of >18.25 MΩ·cm was used for the experiments.

### 2.2. Preparation of Graphene Oxide (GO)

GO was prepared from natural graphite powder using the improved Hummers method. The detailed preparation method is described in our previous report [28].

### 2.3. Preparation of FGO

First, 0.2 g graphene oxide powder was added to 250 mL ultrapure water and stirred for 120 min under ultrasonic dispersion. This yielded a GO dispersion with concentration of 0.8 mg·mL^−1^. Secondly, 0.4 g PPD was added to the GO dispersion, followed by 10 min of ultrasound mixing. Next, the solution was poured into a 500 mL 3-neck boiling flask and magnetically stirred in a water bath with refluxing at 10 °C for 24 h. Subsequently, the solution was filtered off using polypropylene (PP) thin film with mean pore size of 0.2 μm, and washed five times with ethanol and ultrapure water. Finally, the PP thin film was dried at 80 °C for 24 h to obtain the product. The above processes were then repeated by changing the reaction temperature to 30, 50, 70, and 90 °C, respectively. The composite thin films obtained at different temperatures were denoted as, FGO-T (T = 10, 30, 50, 70, and 90), where T represents the temperature. All samples were immersed in methanol solution for 10 h and then dried at 80 °C for 2 h, resulting in samples denoted as FGOS-T (T = 10, 30, 50, 70, and 90).

### 2.4. Characterization

Nicolet-5700 infrared spectrometer (FT-IR, Thermo Nicolet Corporation, Madison, WI, USA) with a scanning range of 4000~500 cm^−1^ was used for bonding characterization using the KBrpellet method. XSAM800 multifunctional electron spectrometer was employed for surface analysis (XPS, Kratos Company, Manchester, UK), with Al target (1486.6 eV) and X-ray gun (12 KV × 15 mA, in FAT mode). The data were corrected using carbon contamination C1s, X’pert MPD Pro X-ray diffractometer (XRD, PANalytical B.V., Almelo, The Netherlands), with Cu target, DS: (1/2)°, SS: 0.04 rad, AAS: 5.5 mm, at the scan range from 5° to 45°. Raman spectroscopy was performed on a Renishaw InVia spectrograph (Wharton Andech, UK), with Ar^+^ excitation source, wavelength of 514.5 nm, and scanning range from 400 to 4000 cm^−1^. Microstructure analysis was conducted using Ultra 55 field emission scanning electron microscope (FE-SEM, Zeiss Instruments, Stuttgart, Germany).

## 3. Results and Discussion

### 3.1. Structural Changes of FGO at Different Reaction Temperatures

**Figure 1:**
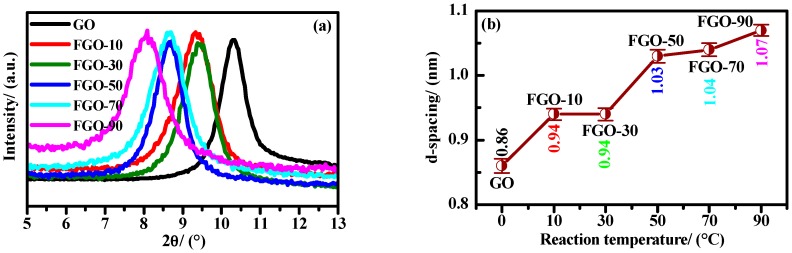
XRD patterns (**a**) and d-spacing (**b**) of GO and FGO-T samples.

Figure 1 shows the XRD patterns and interlamellar spacing *d* values of GO and FGO-T. The *d* value of GO was determined as 0.86 nm while the *d* values of FGO-T (T = 10, 30, 50, 70, and 90) were 0.94, 0.94, 1.03, 1.04, and 1.07 nm, respectively. Compared to GO, the *d* value of FGO-T was larger and increased with the reaction temperature, showing different connection modes between PPD and GO at various temperatures. According to the results of Lu et al. [29], the XRD data and spacing values indicated that the PPD molecules were grafted into the layers of GO.

**Figure 2:**
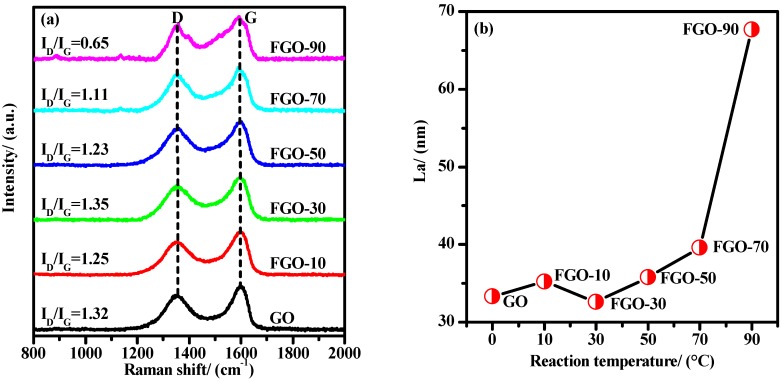
Raman spectra (**a**) and L_a_ values (**b**) of GO and FGO-T samples.

Figure 2 depicts the Raman spectra of GO and FGO-T. In the first order Raman spectral region, both samples showed two major characteristic peaks at 1353 and 1599 cm^−1^, corresponding to the D and G bands of graphene oxide structures, respectively. The D peak was attributed to the double resonance Raman scattering process near critical point K at graphene Brillouin zone, indicating the presence of structural defects [30], while the G peak was caused by E_2g_ eigen vibration of sp^2^ carbon domains [31]. As shown in Figure 2a, the G peaks of FGO-T were shifted from 1585 to 1599 cm^−1^, suggesting that oxidation and PPD caused changes in the graphene structure. The ratio of the integral intensity between the D and G peaks (I_D_/I_G_) determines the disorder level in crystal structure [32]. With the increase in reaction temperature, the value of I_D_/I_G_ basically presented a decreasing trend, indicating that the structural order in samples was partially restored.

Furthermore, it has been empirically found that the disorder decreases the *sp^2^* plane domain (L_a_) in product structures [33].The L_a_ value can be obtained with the formula of L_a_ = (2.4 × 10^−10^) × λ^4^_laser_ (I_D_/I_G_)^−1^, where λ_laser_ is the excitation wavelength [34]. As shown in Figure 2b, compared to GO, the L_a_ value of FGO-T generally increased, indicating that addition of PPD molecules increased the order and the *sp^2^* plane in GO was restored gradually. Moreover, from 10~70 °C, the change in L_a_ values was relatively small, while over 70 °C, the change in L_a_ was the largest. This indicated that elevated temperature could promote the amidation reactions, and the optimum temperature for amidation reaction should be above 70 °C [35].

### 3.2. Effect of Reaction Temperature on FGO Appearance

**Figure 3:**
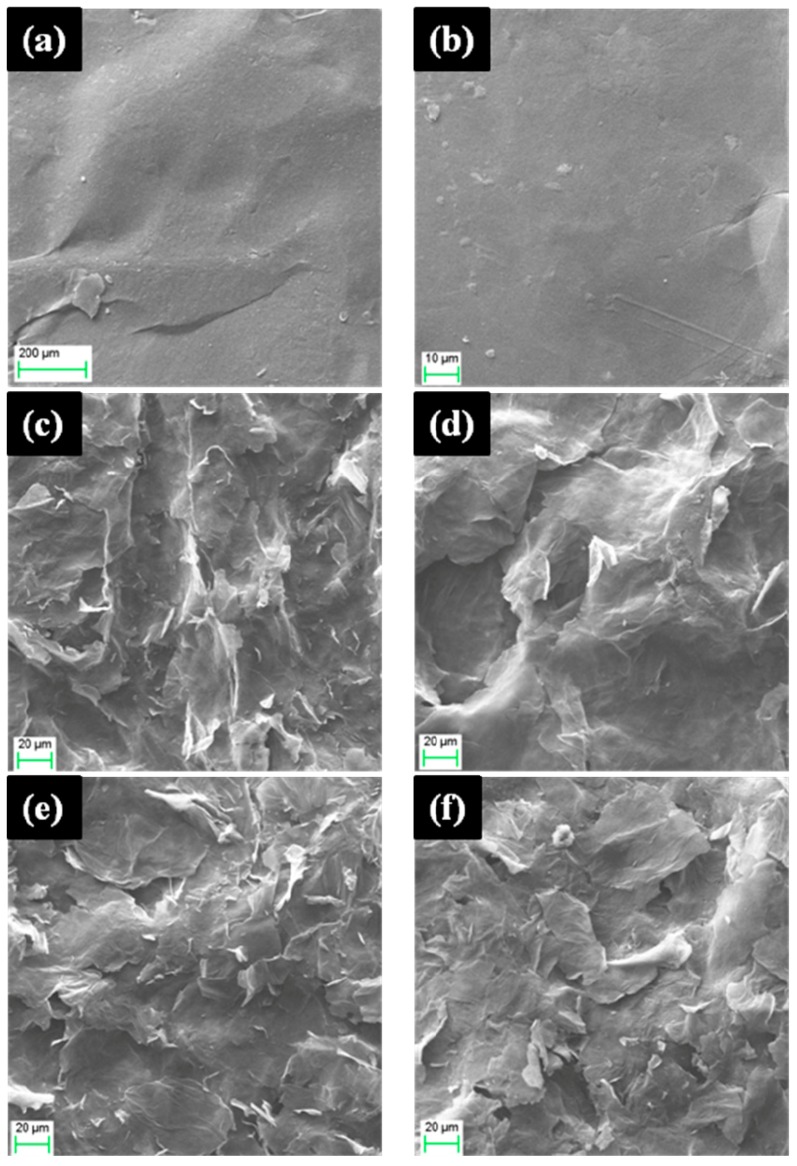

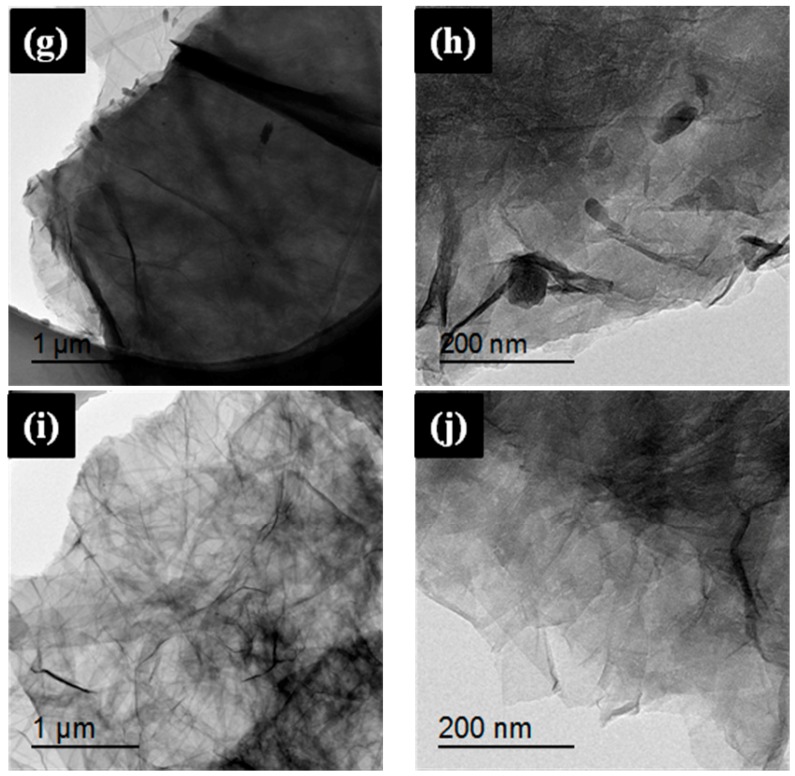
SEM images of (**a**,**b**) GO; (**c**) FGO-10; (**d**) FGO-50; (**e**) FGO-70; and (**f**) FGO-90; TEM images of (**g**,**h**) FGO-50 and (**i**,**j**) FGO-90.

The morphologies of GO and FGO-T (T = 10, 50, 70, and 90) are shown in Figure 3. The most significant difference between GO and FGO-T is their surface roughness. The GO surface appeared flat and smooth (Figure 3a,b). However, as shown in Figure 3c–f, when the GO interacted with PPD, the surface of FGO-T films became rough. PPD is a rigid structure and acts as a nanospace barrier, blocking the stacking of GO sheets. Therefore, the surface of FGO became rough and showed more cracks. The surface roughness was similar to that reported by Shanmugharaj et al. [23] for composites which were prepared by the reaction between GO and alkylamines of varying chain lengths. However, the effect of temperature on surface roughness was less than that of chain lengths. As the temperature increased, the surface roughness of FGO-T films became smaller. This can be attributed to the fact that the elevated temperature promoted amidation reactions instead of adsorption.

Figure 3g,h presents the TEM images of the FGO-50 sample. It can be seen clearly that some PPD particles were wrapped within or on the surface of graphene sheets. With the increase in reaction temperature (Figure 3i,j), the adsorbed PPD particles disappeared. The FGO-90 sample presented a wrinkled and transparent nanosheet with a lateral dimension of about several micrometers. The TEM results are consistent with the SEM data.

In solution, the COOH at the edge of GO nanosheet will be deprotonated, making its lamellae negatively charged [36]. Due to the electrostatic repulsion between adjacent sheets and a large number of hydrophilic oxygen-containing functional groups, the GO can be dispersed steadily in water. This feature can be expressed by Equation (1):

R–COOH ⇋ R–COO^−^ + H^+^ (1)

After addition of PPD molecules, the activity of NH_2_ in PPD molecule decreased at low temperatures. The attachment of PPD molecules between GO slice layers increased due to physical adsorption. On the other hand, in solution, some PPD molecules were protonated according to Equation (2):

R–NH_2_ + H_2_O ⇋ R–NH_3_^+^ + OH^−^ (2)

Equations (1) and (2) indicate that when H^+^ combines with OH^−^, water molecule will be produced and the forward reaction will be favoured. Therefore, most GO slices will be negatively charged while protonated PPD molecule will be positively charged. Then, both will be connected through ionic bonding. In addition, the non-protonated NH_2_ will connect with some oxygen-containing groups though non-covalent hydrogen bonding. With increase in reaction temperature, the activity of NH_2_ in PPD molecule will grow, and the ionic bonding between PPD and GO will strengthen. This would result in stacked and compact GO slices.

At the reaction temperature of 90 °C, the sample surface contained numerous pits but less scattered pieces. Compared to FGO-T at low temperature, the GO slice surface appeared more flat and smooth. This was probably due to the higher activity of –NH_2_ in PPD molecule, which caused large amounts of PPD molecules to covalently react with the oxygen-containing groups in GO. This lowered the space barrier role of PPD for GO slices. However, some GO slices were cross-linked with PPD monomer, making the connection between slices more tight and smooth, hence increasing the order of the structure.

### 3.3. Influence of Temperature on Oxygen-Containing Functional Groups and Types of Interactions

**Figure 4:**
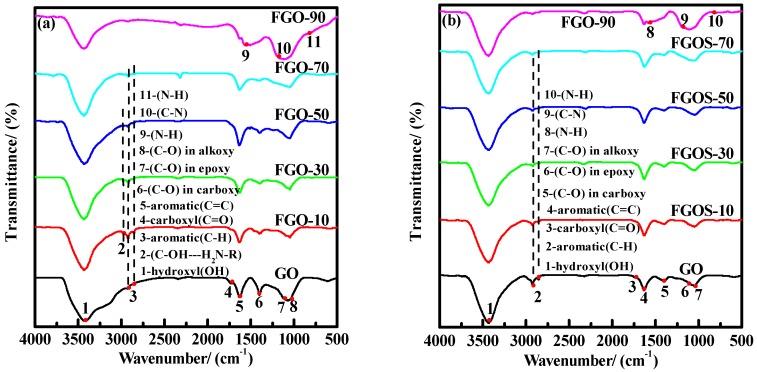
FT-IR spectra of GO and FGO-T samples: (**a**) without and (**b**) with methanol soaking.

Figure 4 shows the FT-IR spectra of GO and FGO-T before and after soaking in methyl alcohol. Numerous oxygen-containing groups were present in GO (Figure 4a). The absorption peaks at 3431, 2922, 2845, 1731, 1625, 1400, 1096 and 1038 cm^#x2212;1^ were attributed to the stretching vibration of hydroxyl (OH), CH_2_ anti-symmetry and symmetry in benzene ring framework, carbonyl group (C=O), benzene ring skeleton (C=C), carboxyl group (O–C=O), epoxy group (C–O–C) and alkoxy group (C–O), respectively [37,38].

By contrast, FGO-T (T = 10, 30, 50, and 70 °C) showed new absorption peaks at 2982 cm^−1^ and 833 cm^−1^, which can be ascribed to the hydrogen-bond interaction between –NH_2_ and oxygen-containing group in GO, and N–H bending vibration in PPD, respectively [39]. The new absorption peaks of FGO-90 at 1583 and 1180 cm^−1^ were ascribed to N–H stretching vibration and C–N stretching vibration, respectively [27,36].These results indicate the occurrence of covalent reaction between PPD molecule and GO at 90 °C. 

**Figure 5:**
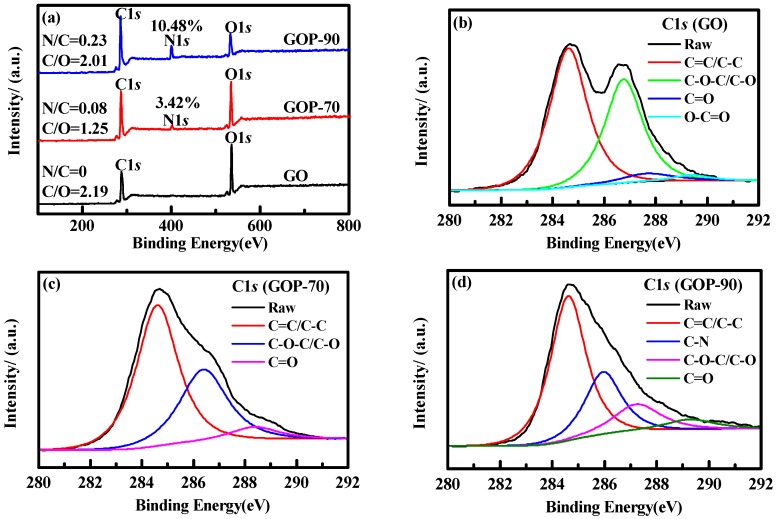
XPS data of GO and GOP samples: (**a**)contents of C, N and O, (**b**) C1s spectrum of GO, (**c**) C1s spectrum of GOP-70, and (**d**) C1s spectrum of GOP-90.

Therefore, according to SEM analyses, the type of bond that would allow the PPD monomer to attach onto GO at temperatures from 10~70 °C can only be based on physical adsorption: (I) hydrogen-bond (C–OH…H_2_N–X) between oxygen-containing group of GO and NH_2_ group of PPD molecule, and (II) ionic bond (–COO^−^H_3_^+^N–R) between protonated PPD and weakly acidic GO. However, above 90 °C, NH_2_ in PPD reacted with the oxygen-containing group of GO, resulting in C–N bond (Figure 5).

To further confirm the above hypothesis, FT-IR analysis of FGOS-T was performed and the results are presented in Figure 4b. Compared to Figure 4a, the absorption peaks of FGO-T (T = 10, 30, 50, 70) at 833 and 2982 cm^−1^ vanished. These peaks were ascribed to N–H bending vibration with hydrogen bonding between –NH_2_ in PPD and the oxygen-containing group of GO, respectively. The absorption peaks of FGOS-90 at 1583, 1176 and 834 cm^−1^ attributed to N–H stretching vibration, C–N stretching vibration and N–H bending vibration, respectively, were consistent with those observed for FGO-90. In addition, compared to GO, the absorption peaks caused by O–C=O and C–O–C stretching vibrations vanished from the spectra. Therefore, it can be concluded that from 10~70 °C, PPD monomer interacted with GO through non-covalent hydrogen and ionic bonds. After soaking in methyl alcohol, the physical adsorptions in PPD were removed. At 90 °C, PPD monomer covalently reacted with GO, resulting in a C–N covalent bond that was still intact after being soaked in methyl alcohol.

To further verify the proposed hypothesis, XPS was performed on GO, FGO-70, and FGO-90 (Figure 5a). GO showed two spectral peaks at 286.0 and 535.0 eV, corresponding to C1s and O1s peaks, respectively [25,28]. By contrast, FGO-70 and FGO-90 displayed not only C1s and O1s peaks but also a new N1s peak near 401 eV, indicating the presence of N atom [27,36]. In addition, the relative percentage contents of N atom were estimated to be 3.42% and 10.48%, suggesting that N content in FGO-90 was higher than that in FGO-70. Figure 5b–d shows the peak-differentiating of GO, FGO-70, and FGO-90. GO revealed characteristic peaks at 284.6, 286.7, 287.9 and 289.4 eV, corresponding to C=C/C–C, C–O–C/C–O, O–C=O and C=O, respectively. FGO-70 displayed characteristic peaks of C=C/C–C, C–O–C/C–O, and C=O. Finally, FGO-90 showed not only the above characteristic peaks but also a new peak at 285.9 eV, ascribed to C–N bond [28,36].

**Figure 6:**
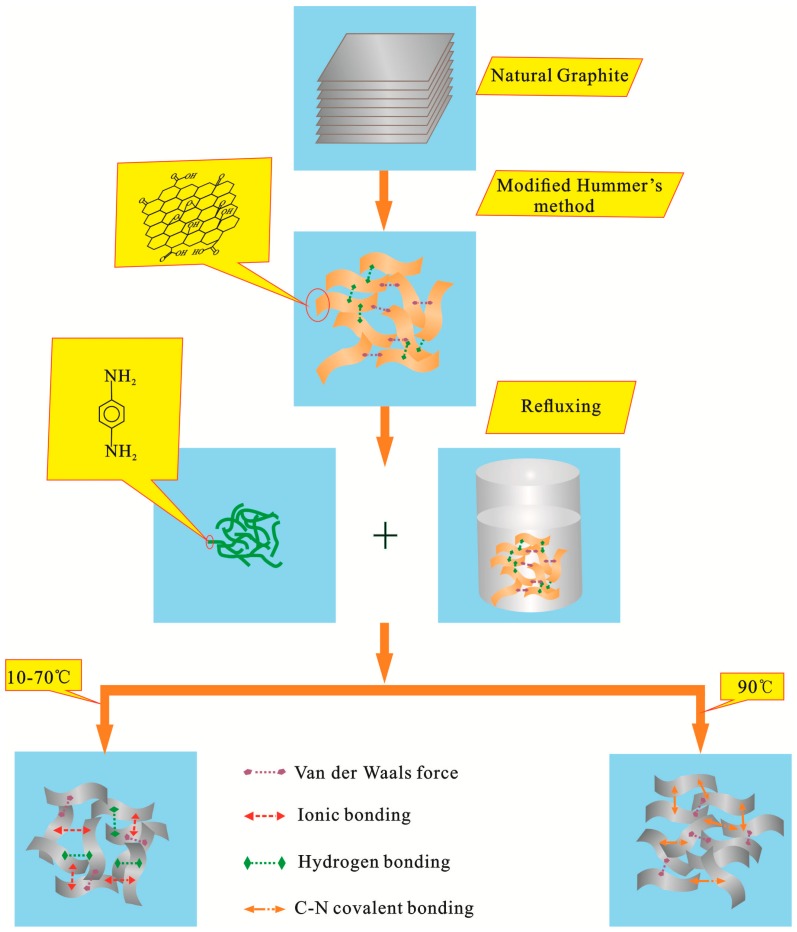
Schematic representation of reaction between GO and PPD.

The above analyses confirmed the absence of covalent C–N bond in FGO-70. In other words, from 10~70 °C, only non-covalent bonding existed between PPD and GO. However, covalent C–N bond formed at 90 °C. FT-IR and XPS data showed that the carboxyl (O–C=O) group in GO was removed by epoxy group (C–O–C) during the reaction process. This indicated that PPD preferred to react first with the carboxyl group and then with the epoxy group. The results of this study were slightly different from a previous report on amidation reactions. Xue et al. [40] found that the grafting of ethylenediamine on the surface of GO can be carried out under very mild conditions, even at 273 K, and the amine was grafted on the surface of GO mainly by a nucleophilic ring opening reaction between the amine and the epoxy group of GO. This may be explained as follows. Previous studies have suggested that benzene ring in PPD and its amine lone pair possess high resonance stability [25,28,36], reducing the reactivity of amine with epoxy groups. Moreover, the PPD’s aromatic ring has large steric hindrance, which would not allow it to either attack the carbon of GO nanosheet or undergo a ring-opening reaction with epoxy at low temperatures. At 90 °C, the amine of PPD showed a higher activity. The NH_2_ in PPD underwent amidation reaction with COOH at edge of GO, and nucleophilic substitution with the surface C–O–C groups. The types of interactions at different reaction temperatures based on SEM, FT-IR and XPS results are summarized in Figure 6.

## 4. Conclusions

In this work, FGO was synthesized via the reaction between GO and PPD through a simple one-step hydrothermal reflux method. The structures, functional groups and compositions of the products were investigated by XRD, Raman, SEM, FT-IR, and XPS analyses. The effects of reaction temperature on the structure of modified composites were also examined. It was found that the surface roughness and the *d* value of the product increased with the rise in reaction temperature. When the reaction temperature was low (10~70 °C), most of the PPD monomer reacted with GO through non-covalent ionic and hydrogen bonds. As the reaction temperature increased, the order of the crystal structure gradually improved. When the reaction temperature was high (>70 °C), grafting of PPD was the primary reaction. PPD was grafted on the surface of GO through covalent C–N bonding by first attacking the carboxyl groups and then the epoxy groups of GO.
